# Identification of Three Cuproptosis-specific Expressed Genes as Diagnostic Biomarkers and Therapeutic Targets for Atherosclerosis

**DOI:** 10.7150/ijms.83009

**Published:** 2023-05-08

**Authors:** Yong-tong Chen, Xuan-hao Xu, Ling Lin, Shuai Tian, Gui-fu Wu

**Affiliations:** 1 Department of Cardiology, the Eighth Affiliated Hospital of Sun Yat-sen University, Shenzhen, Guangdong 518033, China.; 2 Department of Radiology, the Eighth Affiliated Hospital of Sun Yat-sen University, Shenzhen, Guangdong 518033, China.; 3 Guangdong Innovative Engineering and Technology Research Center for Assisted Circulation, Shenzhen, Guangdong 518033, China.; 4 NHC Key Laboratory of Assisted Circulation (Sun Yat-sen University), Guangzhou, Guangdong 510080, China.

**Keywords:** atherosclerosis, cuproptosis, cuproptosis-related gene (CRG), ceRNA network, bioinformatics analysis

## Abstract

Atherosclerosis is a chronic, inflammatory disease characterized by a lipid-driven infiltration of inflammatory cells in large and medium arteries and is considered to be a major underlying cause of cardiovascular diseases. Cuproptosis, a novel form of cell death, is highly linked to mitochondrial metabolism and mediated by protein lipoylation. However, the clinical implication of cuproptosis-related genes (CRGs) in atherosclerosis remains unclear. In this study, genes collected from the GEO database intersected with CRGs were identified in atherosclerosis. GSEA, GO and KEGG pathway enrichment analyses were performed for functional annotation. Through the random forest algorithm and the construction of a protein-protein interaction (PPI) network, eight selected genes (LOXL2, SLC31A1, ATP7A, SLC31A2, COA6, UBE2D1, CP and SOD1) and a vital cuproptosis-related gene FDX1 were then further validated. Two independent datasets (GSE28829 (N = 29), GSE100927 (N = 104)) were collected to construct the signature of CRGs for validation in atherosclerosis. Consistently, the atherosclerosis plaques showed significantly higher expression of SLC31A1, SLC31A2 and lower expression of SOD1 than the normal intimae. The area under the curve (AUC) of SLC31A1, SLC31A2 and SOD1 performed well for the diagnostic validation in the two datasets. In conclusion, the cuproptosis-related gene signature could serve as a potential diagnostic biomarker for atherosclerosis and may offer novel insights into the treatment of cardiovascular diseases. Based on the hub genes, a competing endogenous RNA (ceRNA) network of lncRNA-miRNA-mRNA and a transcription factor regulation network were ultimately constructed to explore the possible regulatory mechanism in atherosclerosis.

## Introduction

Atherosclerosis (AS), a lipid-driven chronic inflammatory disorder of the arteries, is a major cause for the high morbidity and mortality of cardiovascular diseases, such as myocardial infarctions (MI), stroke, and peripheral vascular necrosis 1. Atherosclerotic cardiovascular disease (ACD) is the leading cause of death worldwide. In developing countries, the sanitary and economic burdens of ACD are increasing 2. It has been reported that age, high blood pressure, diabetes, dyslipidemia, obesity and diet, physical inactivity, smoking are common risk factors for atherosclerosis. As for sex, estrogen has cardioprotective effects that decrease cholesterol and LDL, so premenopausal women have a lower risk of atherosclerosis than men 3. Chronic inflammation is the basic pathological basis of atherosclerosis under the mechanism of the accumulation of lipids in arterial wall cells induced by atherogenic LDL in the circulating blood, which activates the pro-inflammatory response when macrophages phagocytosis LDL, intensifying intracellular lipid accumulation 4. Chemical modification of LDL arranges from desialylation to oxidation and peroxidation of the oxidized lipids, to which anti-LDL antibodies had a much higher affinity, thus increasing immunogenicity and atherogenic potential of LDL 5. Besides, mitochondrial DNA (mtDNA) mutations in macrophages could cause mitophagy defects and immune responses, leading to chronic inflammation 4. Previous studies have suggested that oxidative stress (OS) plays a destructive role in atherosclerosis 6. Programmed cell death (PCD) is a central biological process that is dysregulated in atherosclerosis. The connotation of cell death has been expanded beyond apoptosis and necrosis to additional forms, including necroptosis, pyroptosis, autophagy, and ferroptosis 7.

Recent research has identified a new form of copper-dependent cell death called cuproptosis, triggered by excess Cu^2+^ 8. Cuproptosis relies on mitochondrial respiration—copper ions cause abnormal aggregation of lipoylated proteins by directly binding to lipoylated proteins in the tricarboxylic acid cycle (TAC) pathway, and interfere with iron-sulfur cluster proteins in the respiratory chain complex, causing a protein-toxic stress response that ultimately leads to cell death 8.

As an essential micronutrient, copper is necessary for various physiological processes in almost all cell types. In cells, Cu is a double-edged sword: On the one hand, Cu is an essential cofactor for many enzymes. On the other hand, excess copper ions can cause oxidative stress and lead to cell death 9. A study indicated that the serum level of Cu is higher in atherosclerotic patients and it increases with severity of atherosclerosis 10, suggesting that Cu plays an important role in the pathogenesis of atherosclerosis.

However, how cuproptosis regulates the progress of atherosclerosis is currently unclear and still requires further investigation. In this study, datasets were obtained from the Gene Expression Omnibus (GEO). Differentially expressed genes (DEGs) and cuproptosis-related genes (CRGs) were identified through bioinformatics analysis. Receiver operator characteristic (ROC) single factor analyses were performed to evaluate the diagnostic value of the cuproptosis-related biomarkers in atherosclerosis. Three hub genes—SLC31A1, SLC31A2 and SOD1—were ultimately selected and validated. Based on the hub genes, a competing endogenous RNA (ceRNA) network and a transcription factor regulation network were ultimately constructed to explore the possible regulatory mechanism in atherosclerosis. The results may provide a cuproptosis-related theoretical foundation for the development of diagnostic biomarkers and therapeutic targets in atherosclerosis.

## Materials and Methods

### Collection of Datasets and CRGs

**Figure 1:**
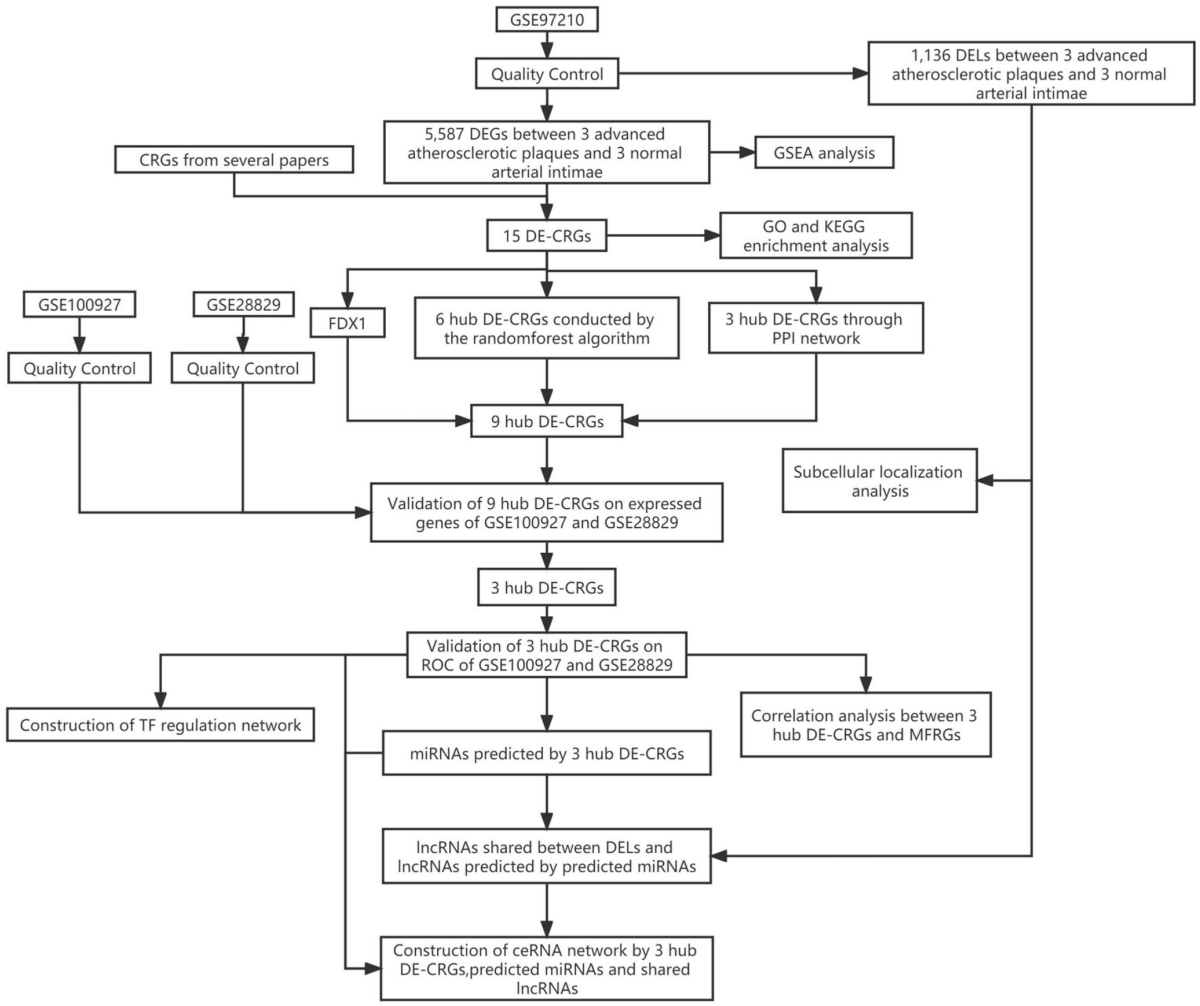
The flow chart of the study design. DELs, differentially expressed lncRNAs; CRGs, cuproptosis-related genes; DEGs, differentially expressed genes; DE-CRGs, differentially expressed cuproptosis-related genes; MFRGs, mitochondrial function-related genes; GSEA, Gene Set Enrichment Analysis; GO, Gene Ontology; KEGG, Kyoto Encyclopedia of Genes and Genomes; PPI, protein protein interaction; TF, transcription factor; ceRNA, competing endogenous RNA.

Training dataset GSE97210(LncRNA/mRNA: n=6, 3 advanced atherosclerotic plaques vs. 3 normal arterial intimae, all males) were collected from the GEO database. Cuproptosis-related genes (CRGs) that promote, inhibit, or mark cuproptosis were retrieved from published articles (PMID: 35298263; PMID: 30706544; PMID: 36226193; PMID: 36119826; PMID: 36253436; PMID: 32570116) 8, 11-15. 44 CRGs were eventually collected for subsequent analyses. A flow chart of the study design is depicted in Figure 1.

### Differentially Expressed Genes and CRGs analysis

Agilent Feature Extraction software 11.0.1.1 was used to analyze acquired array images. Quantile normalization and subsequent data processing were performed using the GeneSpring GX v11.5.1 software package (Agilent Technologies). After quantile normalization of the raw datum, LncRNAs and mRNAs that at least 3 out of 6 samples have flags in present or marginal (all target values) were chosen for further analysis. In R software 4.2.1, the “limma” package was utilized to calibrate the microarray data and identify the DEGs between the atherosclerotic plaques and normal arterial intimae. The “ggplot2” package was utilized to visualize the analysis of differentially expressed mRNAs (DEGs) and differentially expressed lncRNAs (DELs) by the volcano plots (|log2FC| ≥ 1 and p < 0.05). The “ComplexHeatmap” package was utilized to visualize the significantly expressed DE-CRGs in the form of heatmap. The number of DE-CRGs was shown in a Venn diagram using the “Venndiagram” package.

### Gene Set Enrichment Analysis in Atherosclerotic Plaque

Gene set enrichment analysis (GSEA) was employed to detect the related signaling pathways in the progression of atherosclerotic plaque using the OmicStudio online tool. The significant gene sets that conform to the nominal (NOM) p-value < 0.05 and false discovery rate (FDR) < 25% were shown.

### Functional Annotation and Pathway Enrichment of DEGs and DE-FGRs

Gene Ontology (GO) biological process and Kyoto Encyclopedia of Genes and Genomes (KEGG) annotation were performed on the Metascape website. GO and KEGG pathway enrichment analysis using the “ClusterProfiler” package in R was performed to obtain insights into the potential functions of the DE-CRGs. The enrichment results were shown by the GO-KEGG joint FC circle chart. LncRNA enrichment analysis was performed on the LncSEA website, which is a platform for LncRNA related sets and enrichment analysis. The “ggplot2” package in R was used to visualize the LncRNAs localization in cells.

### Screening DE-CRGs through Random Forest algorithm and Construction of Protein-Protein Interaction Network

The “radomForest” package in R was utilized to sort the importance of the DE-CRGs according to the value of MeanDecreaseGini. The STRING database was employed to analyze the interactions among distinct DE-CRGs. The protein-protein interaction (PPI) network was then constructed and visualized in the Cytoscape software 3.8.1. Terms with a combined interaction score > 0.4 are linked by an edge. The top 3 genes of the PPI network were defined as the hub genes, which were calculated based on the maximum neighborhood component (MNC) algorithm with the “cytoHubba” plugin.

### Identification of the Correlation of DRGs and MFRGs

Top 100 genes highly relevant to mitochondrial function were obtained from the MitoCarta3.0 database. The intersecting genes of the DEGs and mitochondrial function-related genes (MFRGs) were defined as the differentially expressed mitochondrial function-related genes (DE-MFRGs). Pearson's correlation analysis between the DEGs and DE-MFRGs was displayed in a heatmap generated by the “ggplot2” package in R.

### Validation of Hub Genes Expression in Atherosclerotic Plaque Datasets

Two microarray datasets of atherosclerotic plaques including GSE28829 (n = 29, 16 advanced vs.13 early plaques) and GSE100927 (n = 104, 69 atherosclerotic lesions, 57 males and 12 females vs. 35 control arteries, 28 males and 7 females) obtained from the GEO database were used to verify the expressions of the hub genes. ROC analyses of the validation datasets were performed by the “pROC” package and data visualization by the “ggplot2” package in R.

### Statistical Analysis

The GEO data matrix has been normalized and conformed to normality test and variance homogeneity test. The “ggplot2” package was utilized to visualize the different expressions of genes by bar charts. Data expressions were presented as mean values ± SEM. Comparisons between the two groups was determined by two-tailed Student's t test. Statistical analyses were performed in R as for individual analysis and statistical significance is displayed as not significant (NS), *p < 0.05, **p < 0.01, and ***p < 0.001.

### Construction of the lncRNA-miRNA-mRNA ceRNA Network and the Transcription Factor Regulation Network

The miRWalk, TargetScan, miRDB and miRTarBase databases were referred to identify miRNA-mRNA pairs. Only miRNAs existed in least two databases and scored =1 or greater were selected. The number of co-expressions of LncRs-predicted and DELs in GSE97210 was shown in a Venn diagram using the “Venndiagram” package. Subsequently, the StarBase dataset was employed to determine the potentially interacting lncRNA-miRNA pairs. lncRNA and mRNA axes with common expression tendencies were selected to construct the final ceRNA networks as miRNA is the intermediate medium. The lncRNA-miRNA-mRNA networks were ultimately constructed and visualized in the Cytoscape software. The TRRUST v2 database was applied to illustrate the roles of the upstream transcription factors on CRGs and to build the transcription factor regulation network.

## Results

### Identification of the DEGs and DE-CRGs in Atherosclerotic Plaques

**Figure 2:**
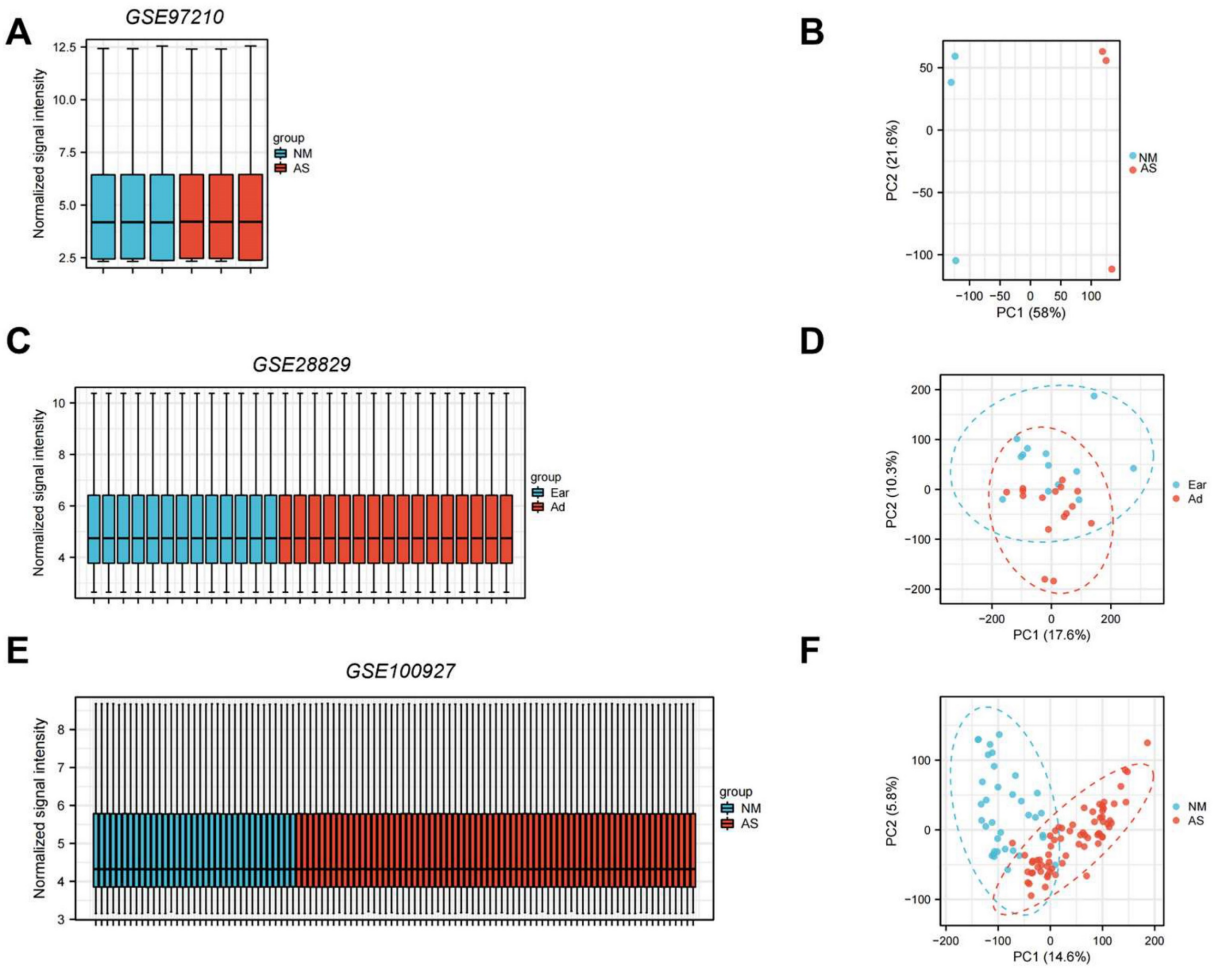
Normalized expression matrices (A,C,E) and PCA diagrams (B,D,F) of the GSE97210, GSE28829 and GSE100927 datasets. PCA, principal component analysis. NM, normal; AS, atherosclerosis.

**Figure 3:**
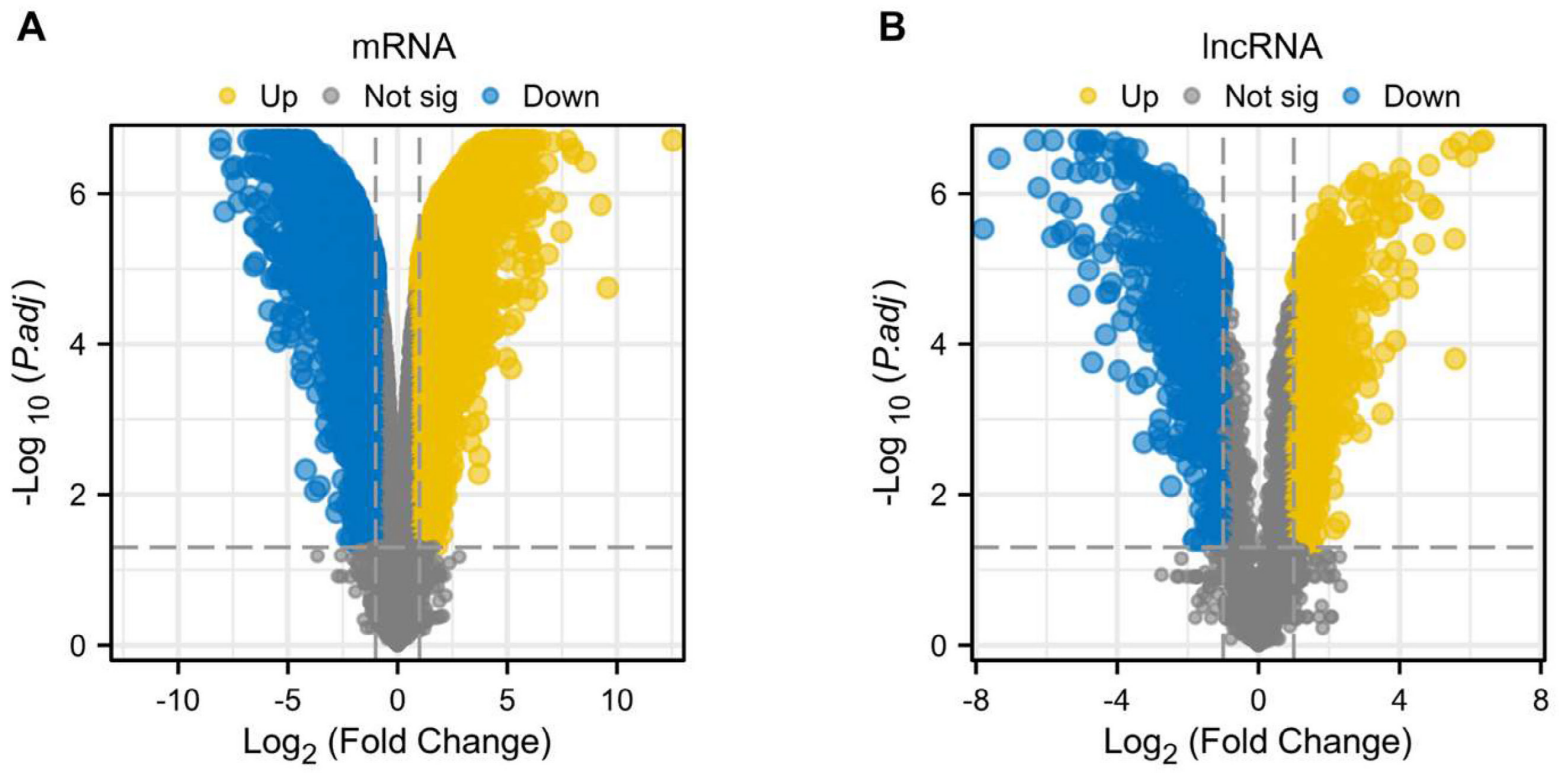
Differentially expressed genes and LncRNAs of the GSE97210 dataset. (A) Volcano plot of DEGs. (B) Volcano plot of DELncRNAs.

**Figure 4:**
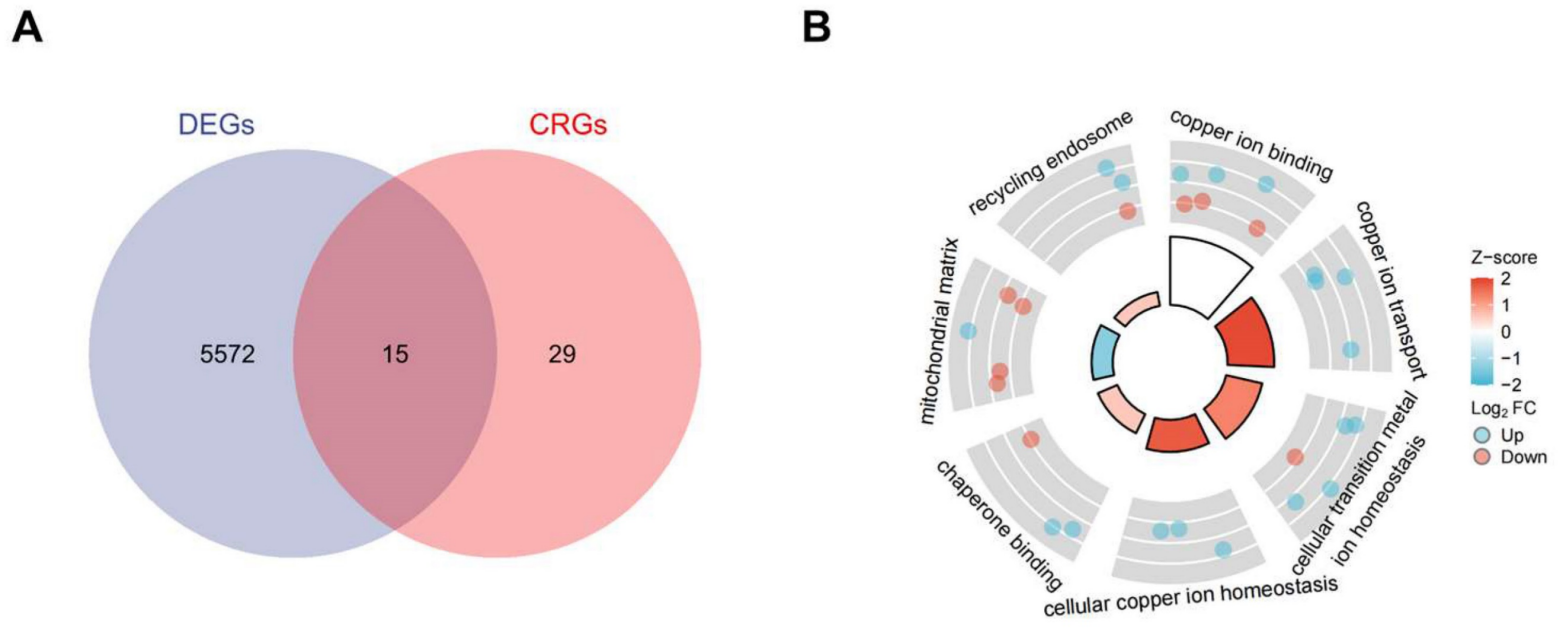
(A) DE-CRGs of DEGs and CRGs by Venn Diagram.DE-CRGs, differentially expressed Cuproptosis-related genes;(B) GO and KEGG combined analysis of DE-CRGs. GO, Gene Ontology; KEGG, Kyoto Encyclopedia of Genes and Genomes.

Standard uniformity and comparability of the gene expression in each sample in the dataset were reflected by box plots (Figure 2A, C, E). The significant differentiation of the clustering among the samples in each dataset was shown by PCA plots (Figure 2B, D, F). Through differential expression analysis of GSE97210, 5,587 genes and 1,136 lncRNAs were significantly differentially expressed in the atherosclerotic plaques compared with the normal intimae, with the thresholds of |log2FC| ≥ 1 and p < 0.05 (Figure 3). 44 CRGs were collected to investigate the CRGs differentially expressed in the atherosclerotic plaques. After taking the intersection of the DEGs and CRGs, a total of 15 differentially expressed CRGs were defined as differentially expressed cuproptosis-related genes (DE-CRGs) (Figure 4A).

### Functional Enrichment Analysis of DEGs and DE-CRGs

**Figure 5:**
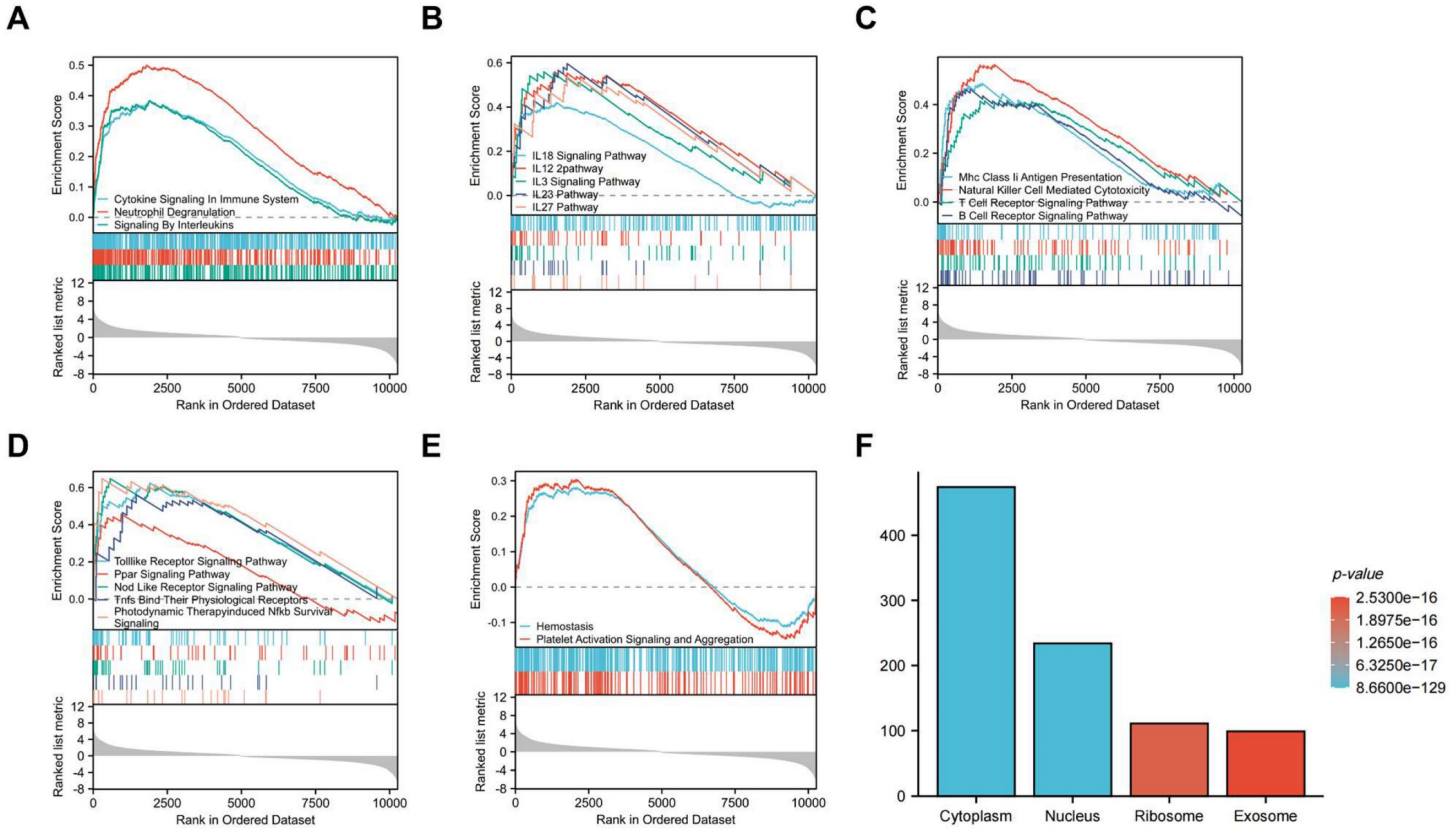
GSEA analysis of CRGs and enrichment analysis of DELs. (A) Inflammatory cells related items. (B) Interleukin related items. (C) lmmune cells related items. (D) Inflammatory and immune-related classical signaling pathways. (E)Coagulation related items. (F)Cell localization analysis of DELs.

Gene Set Enrichment Analysis (GSEA) was subsequently conducted to identify the distinct pathways of DEGs in the atherosclerosis plaques. Results showed that DEGs mainly enriched in inflammation and immunology aspects, especially manifesting in neutrophil degranulation, IL23 pathway, photodynamic therapy induced Nfkb (the nuclear factor kappa B) survival signaling and natural killer cell mediated cytotoxicity (Figure 5A, B, C, D). As inflammatory and immune-related classical signaling pathways, Toll like and Nod like receptor signaling pathways also revealed high DEGs enrichment (Figure 5D). Moreover, high enrichment scores were presented in coagulation system, especially hemostasis as well as platelet activation signaling and aggregation in the atherosclerotic plaques (Figure 5E). LncRNA enrichment analysis on LncSEA website showed that LncRNAs were chiefly located in cytoplasm, with the ratio of cytoplasm and nucleus about 2:1 (Figure 5F). There were 474 LncRNAs located in cytoplasm, followed by 234 LncRNAs in nucleus, 111 LncRNAs in ribosome and 99 LncRNAs in exosome. It is known that cytoplasmic lncRNAs play nonnuclear roles including translational control, cellular metabolism, and signal transduction 16. But cytoplasmic LncRNAs have recently been reported to interact with the ribosome. The RNA degradation in the ribosome may execute a crucial role as the endpoint of cytoplasmic LncRNAs, but the specific mechanisms and biological significance need to be further explored 17. GO and KEGG pathway combined enrichment analysis indicated that 15 DE-CRGs were mainly involved in the binding, transport and cellular homeostasis of copper ion, cellular transition homeostasis of metal ion, chaperone binding, recycling endosome and mitochondrial matrix aspects (Figure 4B). Up-regulated DE-CRGs were mostly enriched in the aforementioned first six aspects with positive correlation, suggesting a facilitating role in cuproptosis. Down-regulated DE-CRGs were mainly involved in mitochondrial matrix aspect with negative correlation, implying cuproptosis inhibition.

### Correlation Analysis and Random Forest Screening of Candidate Cuproptosis-Related Biomarkers

**Figure 6:**
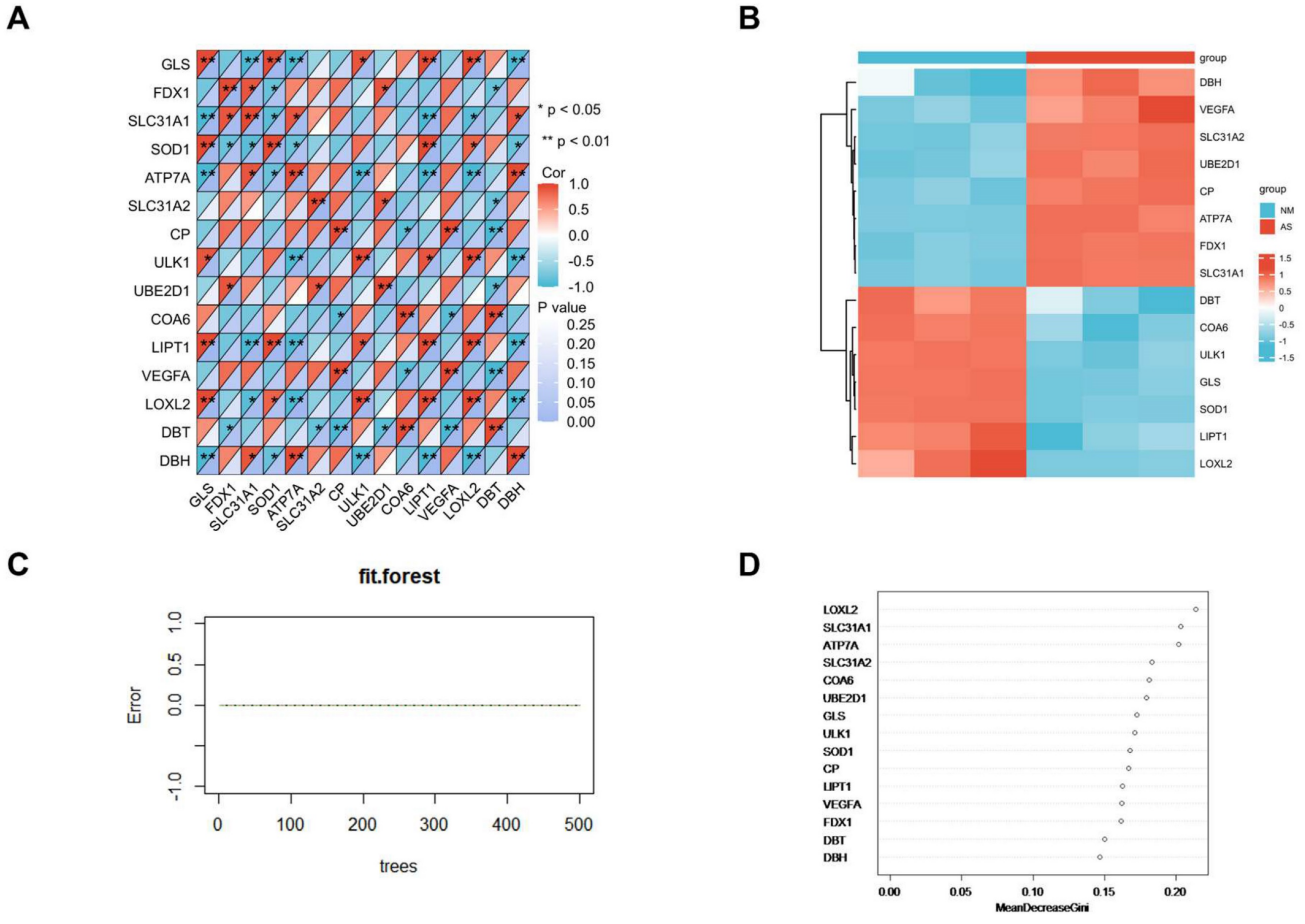
(A)Correlation analysis of the 15 DE-CRGs; (B)Heatmap of 15 the DE-CRGs; (C)Accuracy of the random forest algorithm; (D)Importance order sorted by the MeanDecreaseGini of the 15 DE-CRGs.

Correlation analysis of candidate cuproptosis-related biomarkers showed significant positive correlation in the GSE97210 dataset (Figure 6A), suggesting that these candidate cuproptosis-related biomarkers had synergistic interaction on expression. The expressions of the 15 DE-CRGs in the atherosclerosis and the control groups were visualized in a heatmap, showing that 8 up-regulated DE-CRGs and 7 down-regulated DE-CRGs in the AS group (Figure 6B). The random forest algorithm was used to select important DE-CRGs. According to the Gini index of each candidate cuproptosis-related biomarker, top six of them with strong importance were chosen for further validation. In GSE97210, the final prediction accuracy of the random forest model was 100%. The top six important cuproptosis-related biomarkers were LOXL2, SLC31A1, ATP7A, SLC31A2, COA6, UBE2D1(Figure 6C,6D).

### Protein-Protein Interaction Network Construction and Visualization

**Figure 7:**
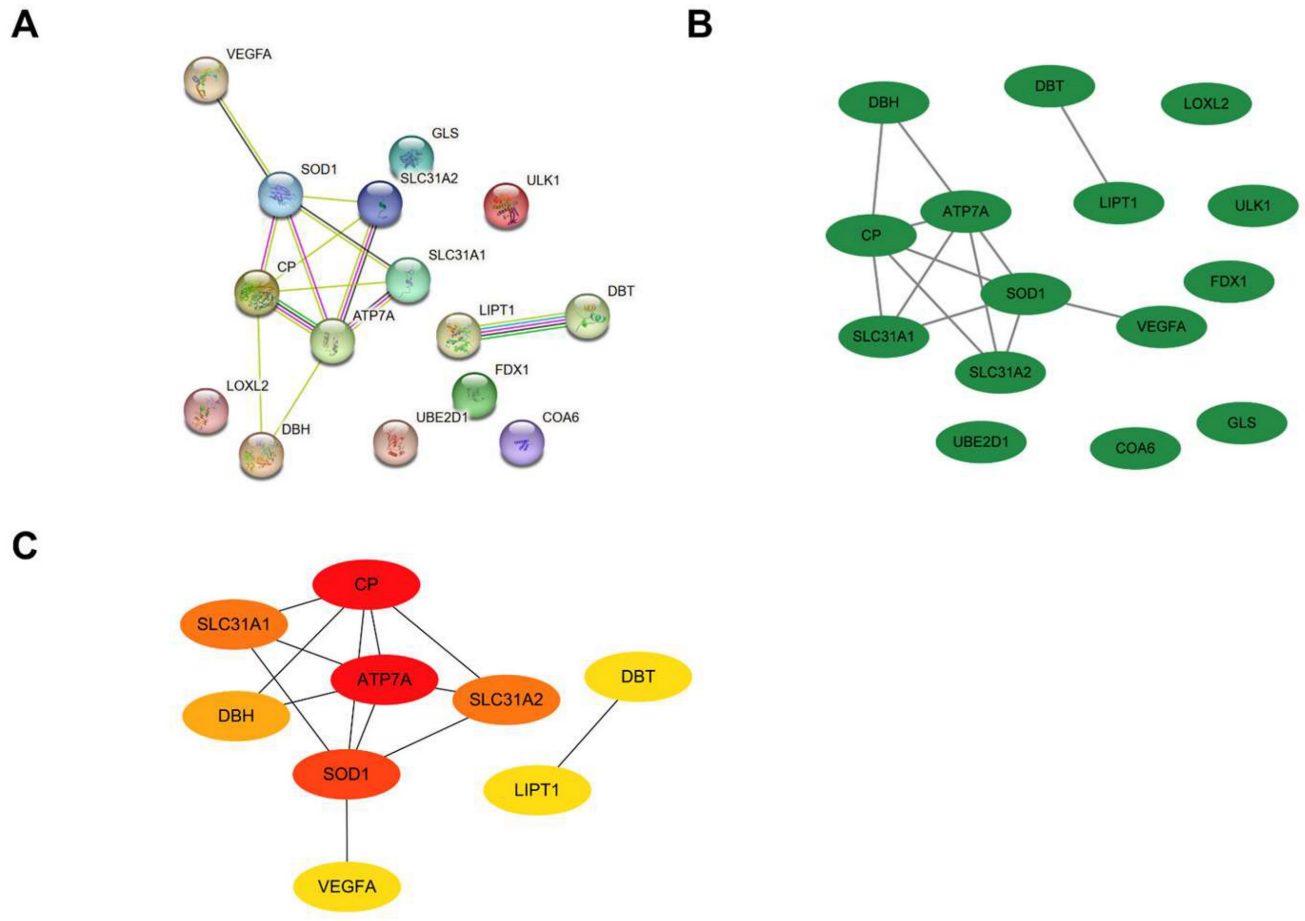
(A)Establishment of PPI network on the STRING database. PPI, protein-protein interaction. (B)Another form of PPI network shown by the Cytoscape. (C)Screening of hub genes by the MNC algorithm.

All of the DE-CRGs were submitted to the STRING database to explore the protein-protein interaction (PPI)network (Figure 7A). Except for the isolated DE-CRGs, the PPI networks of DE-CRGs were displayed including 15 nodes and 13 edges (Figure 7B). According to the scores calculated by the MNC algorithm in the “cytoHubba”, the PPI networks were divided into two clusters. The first cluster composed of seven genes (CP, ATP7A, SOD1, SLC31A1, SLC31A2, DBH, and VEGFA), whereas the second cluster consisted of two genes (DBT and LIPT1). Subsequently, the 3 intersecting genes with top scores (CP, ATP7A and SOD1) were selected as the main genes based on the MNC algorithm (Figure 7C). FDX1 is known to be an important gene involved in the mechanism of cuproptosis 8. Nine DE-CRGs (LOXL2, SLC31A1, ATP7A, SLC31A2, COA6, UBE2D1, CP, SOD1 and FDX1) were finally determined for the validation dataset, combined with the six important DE-CRGs biomarkers identified by the random forest model, the 3 main genes by PPI network and the vital cuproptosis-related gene FDX1.

### Validation of the DE-CRGs Expression in Atherosclerotic Plaques

**Figure 8:**
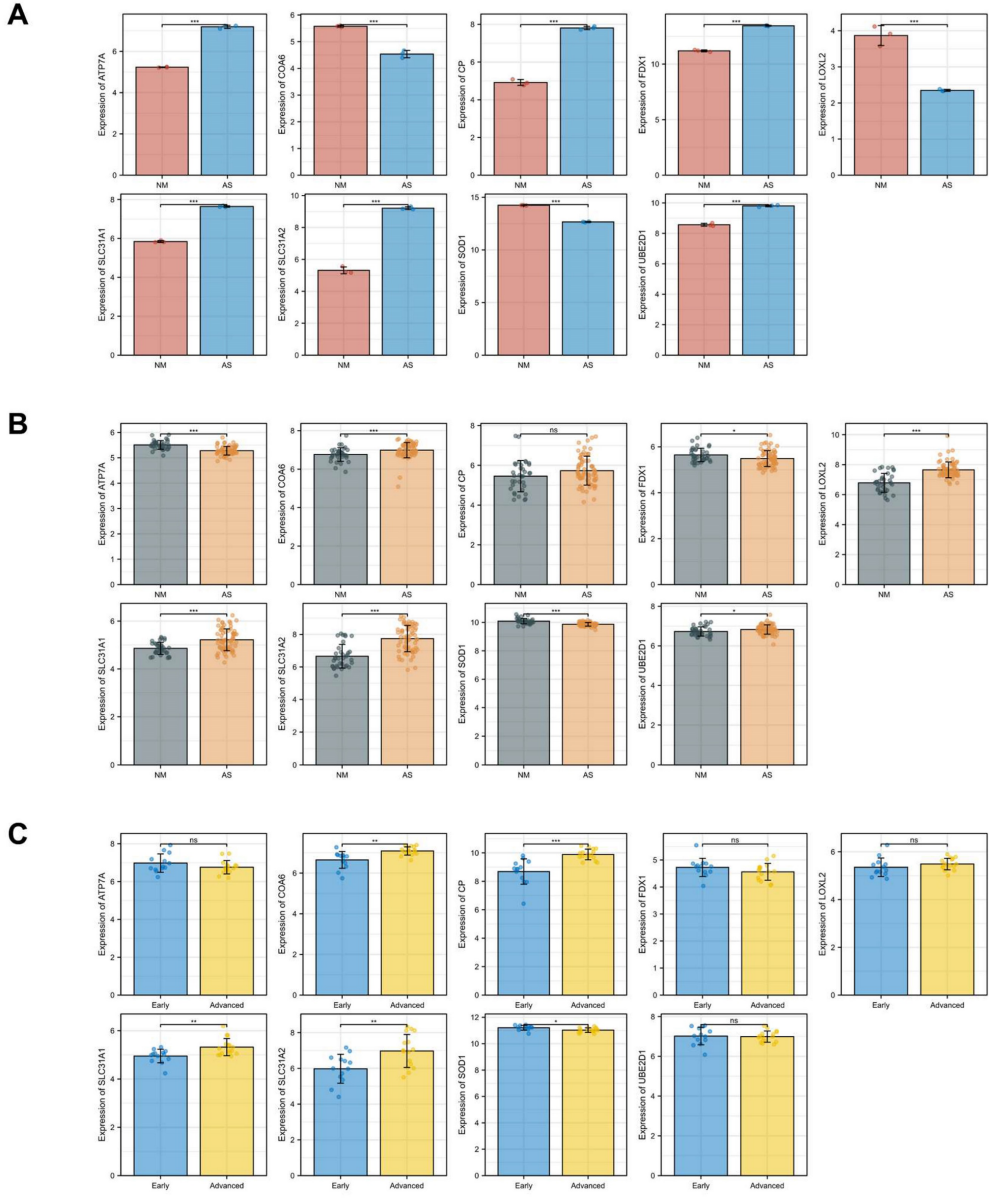
Expressions of the candidate genes in GSE97210(A), GSE100927(B) and GSE28829(C). The numbers above each bar are mean *p* values calculated by students t-test. Data are presented as mean values ± SEM. Comparisons between the two groups was determined by two-tailed Student's t test and statistical significance is displayed as not significant (NS), *p < 0.05, **p < 0.01, and ***p < 0.001.

Another two microarray datasets GSE28829 and GSE100927 were obtained to analyze the differential expression of core genes in atherosclerotic plaques. In the training dataset GSE97210, SLC31A1, ATP7A, SLC31A2, UBE2D1, CP and FDX1 were highly expressed while LOXL2, COA6 and SOD1 were lowly expressed in the atherosclerosis group as showed in the bar charts (Figure 8A). In the validation dataset GSE100927, SLC31A1, SLC31A2, UBE2D1 and SOD1 showed significant expression of differences with consistency in GSE97210 (p < 0.05). In another validation dataset GSE28829, SLC31A1, SLC31A2, CP and SOD1 demonstrated significant expression of differences with consistency in GSE97210 (p < 0.05) (Figure 8C). Although CP had consistent tendency in training and validation datasets, it did not show statistical significance (p = 0.08) in GSE100927 (Figure 8B). COA6 showed significant difference in all three datasets but with opposite trend in validation sets compared with training set. Synthesizing the differential expression results of the two validation datasets, SLC31A1, SLC31A2 and SOD1 were determined as the ultimate hub genes for further validation of the diagnostic value.

### The Diagnostic Value of the Three Cuproptosis-Related Biomarkers in Atherosclerosis

**Figure 9:**
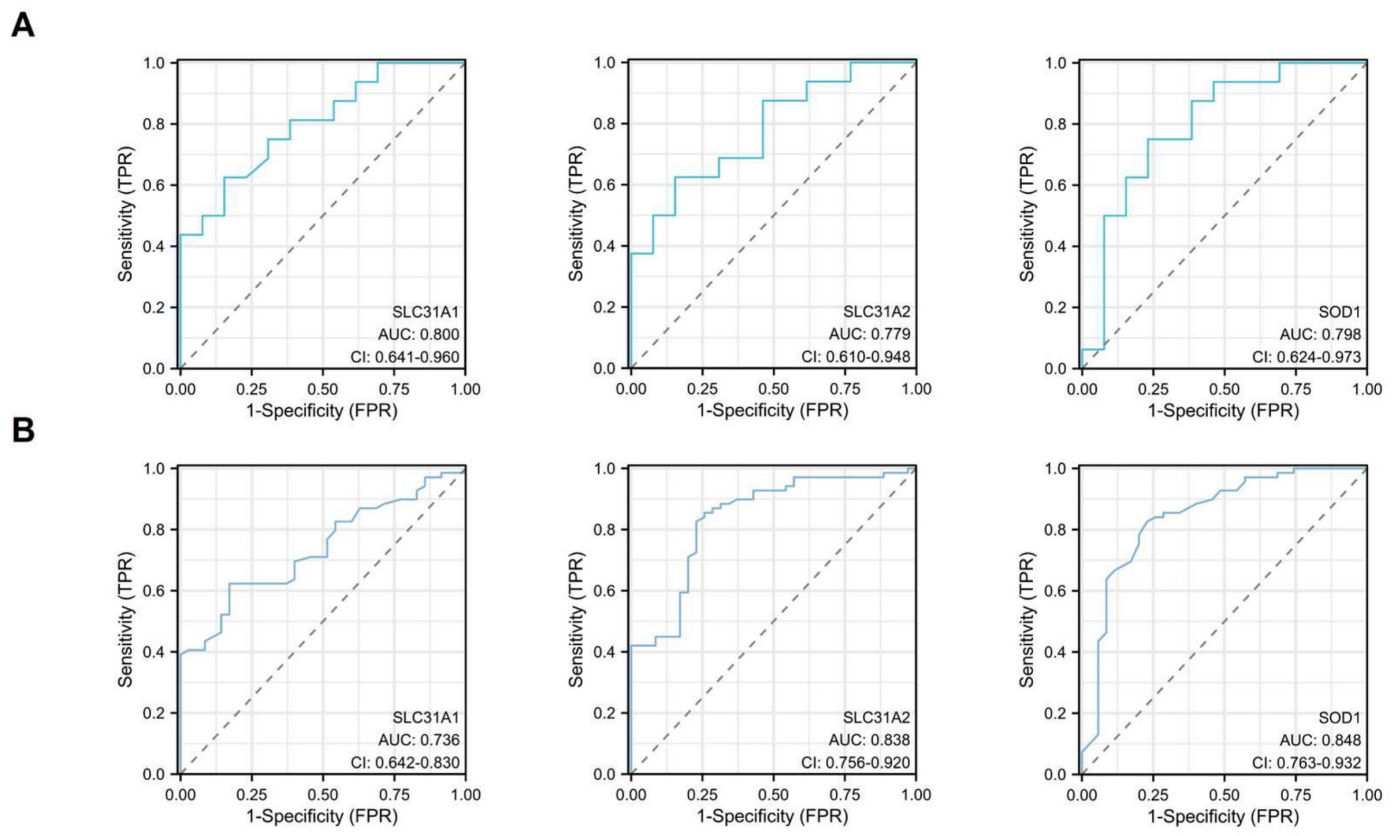
The diagnostic values of SLC31A1, SLC31A2 and SOD1- three hub DE-CRGs biomarkers in AS in the two validation datasets by ROC analysis. (A) GSE28829 (B) GSE100927.

ROC single factor analyses were performed in different datasets to evaluate the diagnostic value of the three hub cuproptosis-related biomarkers in atherosclerosis. The area under curve (AUC) of SLC31A1, SLC31A2 and SOD1 for the diagnosis of atherosclerosis was 0.800, 0.779 and 0.798 respectively in the GSE28829 dataset, with SLC31A1 demonstrating good diagnostic performance (Figure 9A). The AUC of these three cuproptosis-related biomarkers for atherosclerosis was 0.736, 0.838 and 0.848 respectively in the GSE100927 dataset, with SLC31A2 and SOD1 performing good diagnostic value (Figure 9B). Consequently, SLC31A1, SLC31A2 and SOD1 were regarded as three hub cuproptosis-related biomarkers in atherosclerosis.

### Construction of the lncRNA-miRNA-mRNA ceRNA Network

**Figure 10:**
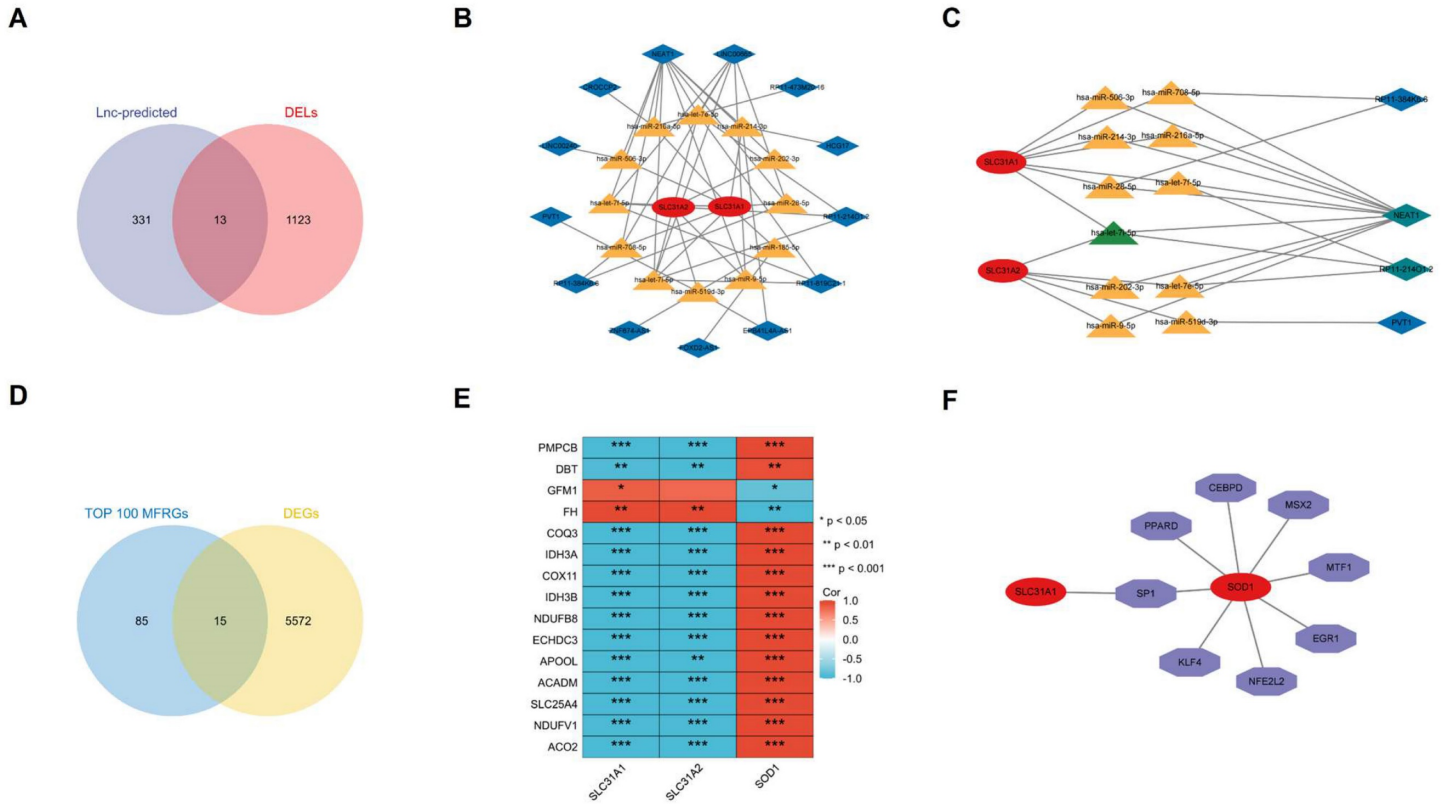
(A)13 common genes between LncRNA predicted and DELs. (B)All possible ceRNA networks. (C)Filtered ceRNA networks. Common molecules were marked in green. (D) DE-MFRGs between TOP 100 MFRGs and DEGs shown by Venn diagram. MFRGs, mitochondrial function-related genes; DE-MFRGs, differentially expressed mitochondrial function-related gene(E)Pearson's correlation heatmap of the relationships among the DE-MFRGs and the three hub DE-CRGs. (F)transcription factor regulation network of the hub DE-CRGs.

The ceRNA regulatory network of lncRNA-miRNA-mRNA was constructed to clarify the potential molecular regulatory mechanisms of these differentially expressed CRGs. Based on the miRWalk, TargetScan, miRDB and miRTarBase reference databases, a group of miRs were predicted by the three hub CRGs. 344 predicted-lncRNAs were predicted by the aforementioned predicted-miRs on the StarBase website. 344 predicted-lncRNAs and 1136 DELs in the GSE97210(|log2FC| ≥ 1 and p< 0.05) were intersected to get 13 shared lncRNAs (Figure 10A). Based on the competitive endogenous RNA hypothesis 18, 19, lncRNA-miRNA-mRNA competing endogenous RNA (ceRNA) networks were constructed to explore the functions of RNAs in atherosclerotic plaques (Figure 10B). Since miRNAs act as intermediate bridges linking mRNA and lncRNA, the ultimate ceRNA networks were constructed considering the consistent expression trend of mRNAs and lncRNAs (Figure 10C). In the ultimate axis, RP11-214O1.2 and NEAT1 as hub LncRNAs, let-7i-5p as hub miRNA, SCL31A1 and SCL31A2 as hub CRGs have multiple cross-regulatory effects. The ultimate ceRNA network contained 4 lncRNA nodes, 11 miRNA nodes, 2 hub gene nodes, and 29 edges.

### Identification of the Relationship Between the Hub CRGs and MFRGs

The relationship between the hub CRGs and the MFRGs was analyzed since cuproptosis is involved in mitochondrial dysfunction 8. Top 100 MFRGs obtained from MitoCarta 3.0, an inventory of mammalian mitochondrial genes, intersected with 5587 DEGs in GSE97210 and then 15 genes were classified as DE-MFRGs (Figure 10D). Pearson's correlation analysis showed that most of DE-MFRGs were highly correlated with the three hub CRGs in the correlation heatmap (|r| ≥ 0.5, p < 0.05) (Figure 10E). Specifically, SLC31A1 and SLC31A2 were particularly positive associated with DE-MFRGs while SOD1 was chiefly negative correlated with DE-MFRGs. It revealed a strong correlation between cuproptosis-related genes and mitochondria, suggesting that CRGs function through mitochondria.

### Construction of the Transcription Factor Regulation Network

The transcription factors (TF) of the three CRGs biomarkers in the TRRUST (version2) database were explored to further clarify the potential mechanism of cuproptosis-related biomarkers in atherosclerosis. The core transcription factors that could regulate CRGs were involved in the regulation network. Two cuproptosis-related biomarkers and eight core transcription factors were displayed by the axis chart (Figure 10F). More specifically, Regulation modes of SOD1 by transcription factors were mainly activation while SP1's mode of regulation of SLC31A1 remains unknown (Table 1).

## Discussion

In the study, we explored the role of cuproptosis in the molecular mechanism of atherosclerosis. Three cuproptosis-related genes—SLC31A1, SLC31A2 and SOD1 were screened out as diagnostic biomarkers for atherosclerosis. Besides, a possible ceRNA network and a transcription factor regulation network were constructed to illustrate molecular mechanism of atherosclerosis.

Atherosclerosis is a chronic inflammatory disease with oxidative stress playing a critical role. Oxidative stress-induced lipid peroxidation has been implicated in the development of atherosclerosis 20. Copper, a strong prooxidant, may play a role in atherogenesis. Experiments revealed that copper causes activation of redox-sensitive transcription factors and upregulation of inflammatory mediators in endothelial cells whereas copper chelation has an inhibitory effect on vascular inflammation and atherosclerotic lesions development 21, 22. Elevated copper levels were detected in the intima of human atherosclerotic lesions 23. Previous studies have found elevated serum copper concentrations to be associated with atherosclerotic disease 24, even cancer and cardiovascular disease 25. It is suggested FDX1 and genes involved in protein lipoylation are hub genes that promote cuproptosis, which are essential for mitochondrial aerobic metabolism 8. In our study, the different expressions of FDX1 in the two validation sets were inconsistent with those in the training set GSE97210, implying that FDX1 may not be an important factor involved in atherosclerosis. Notably, the Cu chaperone ATOX1 and exporter ATP7A were found to be highly expressed and colocalized in neointimal vascular smooth muscle cells (VSMCs) or in the intimal lesions of atherosclerotic vessels 26. ATP7A was found to be important in copper-dependent PDGF-stimulated VSMC migration via recruiting Rac1 to lipid rafts and regulating LOX activity, contributing to neointimal formation after vascular injury 27. It provides insight into ATP7A as a novel therapeutic target for vascular remodeling and atherosclerosis. In our study, ATP7A expressed significant difference in training dataset but failed to prove the diagnostic value of in atherosclerotic plaques validation dataset, which may attribute to insufficient sample sizes. Further experiments are needed to explore the effect of ATP7A in the mechanism of AS. Copper is essential for COX assembly, activity and stability, which is the terminal complex of the mitochondrial electron transport chain. COA6 is an intermembrane space protein that functions in COX biogenesis and activity as well as interacts with other COX assembly factors associated with biogenesis of the Cu_A_ site 28. Studies revealed that COA6 pathogenic mutations were associated with mitochondrial diseases such as hypertrophic obstructive cardiomyopathy, muscular hypotonia and lactic acidosis 29, 30. COX6 expressed significant difference in three datasets but was inconsistent in tendency in our study. Limited by different datasets and sample sizes, the role of COX6 in atherosclerosis needs to be explored. Ceruloplasmin, encoded by CP, is a serum ferroxidase that contains greater than 95% of the copper found in plasma 31. It plays no essential role in the transport or metabolism of Cu. Large clinical studies proved CP association with incident heart failure, mortality, and cardiovascular diseases in the Atherosclerosis Risk in Communities (ARIC) population 32 as well as a direct relationship between ceruloplasmin elevated levels and incidence of coronary heart disease 33. The effect of CP involved in cuproptosis-related atherosclerosis and the functions of ceruloplasmin as a mediator in the development of coronary heart disease need further investigation to explore the possible mechanism.

In our study, we screened three cuproptosis-related genes—SLC31A1, SLC31A2 and SOD1—that are probably implicated in AS via bioinformatics analysis. The members A1 (CTR1) and A2 (CTR2) of the SLC31 solute carriers family belong to a protein network that acts to regulate the intracellular Cu²⁺ concentration within a certain range 34. SLC31A1 predominantly localize in the plasma membrane while SLC31A2 mainly exist in intracellular membranes of the late endosome and lysosome 34. SLC31A1, as coding gene of high-affinity Cu carrier transporter CTR1, is responsible for most of the copper absorption within cells 35. SLC31A1 is necessary for transporting copper to specific organs/tissues and embryonic development in mammals. The specific function of SLC31A2 is not known. It is reported that oxidation of SLC31A1 promotes VEGFR2 internalization and releases signals to enhance angiogenesis, revealing an important mechanism for sensing reactive oxygen species through SLC31A1 to drive neovascularization 36. It is reported that SLC31A1 correlated to Wilson disease, Parkinson's disease, lung and breast cancer 37-40, making it a potential predictor for diagnosis, prognosis and therapeutic target. Expression of the Cu transporter genes ATP7A, ATP7B, SLC31A1 and SLC31A2 were found significantly altered in liver cancer samples with elevated Cu levels 41. Recent study determined prognostic value and immunotherapy guidance of SLC31A2 in lung adenocarcinoma (LUAD) 42. The mechanistic roles of SLC31A1 and SLC31A2 in atherosclerosis and cardiovascular diseases need further experiments to confirm. Within the cytoplasm, Cu transport is closely coordinated by a fine-tuned network of high-affinity Cu chaperones. The chaperone protein CCS delivers Cu to superoxide dismutase 1 (SOD1) to detoxify mitochondria-derived reactive oxygen species (ROS) and maintain Cu homeostasis 26. SOD1 is considered to implicate in several vital biological processes* in vivo*, such as copper homeostasis, apoptosis, angiogenesis and oxidative stress 43. Research revealed that elevated copper in senescent cells is a universal phenomenon and SOD1 presented to increase, accompanied by enhanced anti-oxidative ability by elevated levels of SOD1 44. Copper homeostasis and cellular autophagy proved to have significant connection, both as important facets of age-associated degenerative disease 44. As the results of our study revealed, lower expression of SOD1 in atherosclerosis plaques suggested activated oxidative stress in the process of atherosclerosis. Besides, SOD1 was significantly negatively correlated with DE-MFRGs, supporting the viewpoint that SOD1 inhibited oxidative stress in mitochondria. Analysis of gene and signaling pathways involved in oxidative stress and inflammatory response process regulated by SOD1 demonstrated that SOD1 may affect the heart through myocardial contraction, inflammation, lipid metabolism, and other pathways 45. At present, CRGs have been shown to play a role in a variety of cancers such as pancreatic cancer, breast cancer, colorectal cancer, ovarian cancer 8, 11-15, but there is a lack of relevant research on cardiovascular diseases.

This study is limited in the small sample size in datasets. The training dataset GSE97210 contains 6 samples while validation datasets include 29 samples and 104 samples, respectively. After comprehensive consideration, the training dataset GSE97210 containing data of mRNAs (genes)and LncRNAs was suitable to construct the ceRNA network. Future studies are supposed to increase sample sizes to validate the role of CRGs in atherosclerosis.

## Conclusion

In summary, we identified three hub cuproptosis-related genes—SLC31A1, SLC31A2 and SOD1 and construct a possible ceRNA network and a transcription factor regulation network in the molecular mechanism of atherosclerosis, providing new insights into cuproptosis and atherosclerosis. However, current studies on cuproptosis-related atherosclerosis are at a blank stage. Further explorations are needed to provide more evidence for cuproptosis in the diagnosis and treatment of atherosclerosis.

## Figures and Tables

**Table 1:** TF regulation of hub DE-CRGs.

TF	Target	Mode of Regulation	References (PMID)
SP1	SLC31A1	Unknown	20159940
CEBPD	SOD1	Activation	20385105
EGR1	SOD1	Activation	9867871
KLF4	SOD1	Repression	23370975
MSX2	SOD1	Activation	22824755
MTF1	SOD1	Activation	15378601
NFE2L2	SOD1	Activation	22493435
PPARD	SOD1	Activation	18048767
SP1	SOD1	Activation	8921911;9867871
SP1	SOD1	Repression	11724400

## Abbreviations

AS: atherosclerosis
DEGs: differentially expressed genes
DELs: differentially expressed lncRNAs
CRGs: cuproptosis-related genes
DE-CRGs: differentially expressed cuproptosis-related genes
GSEA: Gene Set Enrichment Analysis
GO: Gene Ontology
KEGG: Kyoto Encyclopedia of Genes and Genomes
MFRGs: mitochondrial function-related genes
DE-MFRGs: differentially expressed mitochondrial function-related genes
PPI: protein protein interaction
ceRNA: competing endogenous RNA
TF: transcription factor
